# Editorial: Advances in the Molecular Biology of Trypanosomatid Pathogens: New Strategies Against Ancient Enemies

**DOI:** 10.3389/fcimb.2021.777008

**Published:** 2021-10-06

**Authors:** Gustavo Benaim, Alberto E. Paniz-Mondolfi, Juan David Ramírez, Emilia Mia Sordillo

**Affiliations:** ^1^ Laboratorio de Señalización Celular y Bioquímica de Parásitos, Instituto de Estudios Avanzados, Caracas, Venezuela; ^2^ Instituto de Biología Experimental, Facultad de Ciencias, Universidad Central de Venezuela, Caracas, Venezuela; ^3^ Department of Pathology, Molecular, and Cell-Based Medicine, Icahn School of Medicine at Mount Sinai, New York, NY, United States; ^4^ Grupo de Investigaciones Microbiológicas-UR (GIMUR), Departamento de Biología, Facultad de Ciencias Naturales, Universidad del Rosario, Bogotá, Colombia; ^5^ Institute for Health Sciences, Mount Sinai St Luke’s & Mount Sinai West, New York, NY, United States

**Keywords:** trypanosomatids, Chagas, leishmaniasis, treatment, pathogenesis, genetics

## Introduction

The Trypanosomatidae emerged as successful parasites of invertebrate and vertebrate hosts hundreds of millions of years ago. (Stevens et al., 2001) Infections by these important human pathogens cause diseases such as sleeping sickness or African trypanosomiasis (*Trypanosoma brucei*), Chagas disease (*Trypanosoma cruzi)* and cutaneous/visceral leishmaniasis that affect millions of people world-wide (Freitas Lidani et al. WHO-Leishmania, 2021). Once almost exclusively considered neglected tropical diseases, transmission of these infections is now recognized in economically-privileged countries such as the United States, which is endemic for McIlwee et al., 2018; WHO-Leishmania, 2021) as well as Chagas disease (Paniz-Mondolfi et al., 2020). The complex and varied life cycles of the Trypanosomatidae, plus their ability to inhabit diverse ecological niches and to infect a wide range of vertebrate and invertebrate hosts have contributed to their persistence and expansion despite efforts toward elimination by the scientific community, global agencies, and governments.

The contributions in this Research Topic utilize genomic, proteomic, transcriptomic and metabolomic methods in combination with traditional epidemiology to provide new insights into the biological mechanisms influencing disease spectrum and progression, pathogenesis, and potential new treatment approaches.

Disease transmission, clinical presentation and progression, and susceptibility to treatment are dependent on the pathogenicity, virulence, tissue tropism, and potential drug sensitivity of the infecting parasite. Several articles in this Research Topic demonstrate the genomic variability among currently circulating strains causing clinical disease and provide evidence for the existence of newly recognized hybrid strains. Luz H. Patino and colleagues assessed the genomic variability of circulating clinical isolates of *L. brasiliensis*, the agent of spundia, from different regions of South America, comparing findings from Columbia and Bolivia to publicly available sequences for isolates from Brazil. Analysis of whole nuclear and mitochondrial maxicircle sequences demonstrated important genomic diversity and evidence of hybridization, indicating genomic plasticity in Leishmania that may be tied to clinical outcome. In their review, Hirotomo Kato and colleagues summarize their recent country-wide studies in Ecuador and Peru; genetic analyses of kinetoplast and nuclear DNA using PCR-RFLP reveal the extent and distribution of *Leishmania* spp. causing infections in these countries. Notably, these authors also describe previously unknown hybrid species that appear to be associated with more severe clinical presentations and altered transmission dynamics. Evidence for genetic exchange in *Leishmania* parasites *in vitro* is provided by Roman Telittchenko and Albert Descoteaux, using promastigote lines of *L. amazonensis* and *L. mexicana* harboring integrated or episomal drug-resistance markers. Genetic exchange is described in both axenic promastigote cultures and infected macrophages. However, growth of the resulting hybrids was not sustained in subcultures, suggesting the recombination was unstable. Variability that can be associated with clinical presentation and outcome is also recognized within *T. cruzi* lineages. In their report, Callejas-Hernández et al. present results from sequencing of mitochondrial maxicircle and minicircle DNA from *T.cruzi* trypomastigotes of the Y and Bug2148 strains, finding conserved motifs in the maxicircles, but unexpected heterogeneity among minicircle sequences. Rusman and colleagues used deep-sequencing of the hypervariable regions of the minicircle kDNAs to highlight a previously undescribed repertoire of guide RNAs in the main lineages of *Trypanosoma cruzi*, demonstrating highly divergent gRNA repertoires among different parasite lineages, and even within those lineages. In addition to variable gRNA class redundancy, these authors found that gRNA classes of different strains may edit mitochondrial mRNAs from other lineages, suggesting a potential biparental inheritance of minicircles. Future studies are needed to shed light on the physiological implications of these findings.

Maldonado et al. reviewed the molecular mechanisms of DNA repair and the functional properties of DNA polymerase Beta-Like enzymes from trypanosomatids. Polymerase beta seems to play a role in kDNA replication as it associates with kinetoplast antipodal sites in some developmental stages in trypanosomatids and are relevant for cell replication. The authors summarized the main characteristics of trypanosomatid polymerase beta like enzymes and the further need to unveil key structural features for a comprehensive understanding of their role in mammal infection.

Understanding biochemical and metabolic events that influence parasite development in the vector and host are also essential to understanding the complexities underlying parasite life cycle, vector competency, host range, and their influence on the spectrum of clinical disease and outcomes. In the comprehensive review from the Meyer-Fernandes group (Freitas-Mesquita et al.), the expression of protein phosphatases in Leishmania parasites was revisited. Phosphatases play a role in adaptation to nutrient starvation during parasite passage through the sandfly midgut, and are important to parasite virulence, *via* modulation of host cytokine production and impairment of the lytic potential of macrophages. Papadaki et al. address the differential gene expression of atypical lipid phosphatases (ALPs), which play a major role in the cell differentiation among *Leishmania* parasites. Focusing on the expression of LDBPK_220120.1 (ALP) in *L. donovani*, the cause of visceral leishmaniasis, these authors report the presence of its gene product in promastigotes and amastigotes, dual specificity for P-Tyr and PI(3)P, the regulatory influence of temperature and pH shifts, and its possible role in the transition between parasite stages to amastigotes in the mammalian cell.

To elucidate the potential impact of the regulation of parasite development in the insect vector on transmission to the mammalian hose, Rolandelli and colleagues assessed immune pathways in Rhodnius prolixus, the triatomine vector of *Trypanosoma rangeli*. These authors detected differential modulation of the insect IMID, toll, and JAK-STAT pathways in insects with or without hemolymph infection in addition to gut infection, presenting an intriguing potential role for transmission to the mammalian host.

Once in a mammalian host, the parasite must establish infection. Ferri and Edreira reviewed the multi-strategic approach by *T. cruzi* to invasion, addressing the several strategies this parasite has developed to disrupt the host cell signaling machinery in order to gain access to the host cell cytoplasm. The authors highlight the role of the parasitophorous vacuole, from which the parasite escapes to the cytoplasm, and wherein differentiation and replication of the parasite take place. In recent years, ingestion of food contaminated by metacyclic trypomastigotes has triggered outbreaks of acute infection of Chagas disease by oral transmission. Muñoz-Calderón et al. characterized the genetic constitution of natural *T. cruzi* populations in individuals who had failed treatment with benznidazole 9 years earlier for infection acquired during an Oral Chagas Disease (OCD) outbreak at a rural school in Venezuela. Observed decreases in parasite loads and population variability indicated by Pre- and Post-Treatment intra-TcI T. cruzi clade I (TcI) diversity suggested the decrease might be a consequence of both natural evolution of the acute infection to the chronic phase and persistence of refractory populations due to treatment selection. One means used by *T. cruzi* to evade host immune response is antigenic variation.

Carlos Talavera-López and colleagues utilized high coverage single-molecule and deep sequence data to analyze genomes from 34 *T. cruzi* clade I (TcI) isolates and clones from different geographic locations against the Sylvio X10/1 TcI reference genome sequence, finding an unusual organizational structure for the region encoding surface molecules. These authors describe synteny of the core genomic regions, but flanked by unique, highly plastic multigene family clusters with abundant interspersed retrotransposons, apparently involved in recombination and generation of antigenic variation. Their comparative genomic analysis of the cohort of clinical TcI strains revealed multiple cases of such recombination events involving surface molecule genes, providing new insights into *T. cruzi* population structure and evasion of the host immune response.

Treatment of trypanosomatid infections remains an ongoing challenge. García-Bustos and colleagues have developed a rapid screening assay utilizing *Leishmania (Leishmania) amazonensis* expressing a red fluorescent protein encoded by pIR1SAT/tdTomato to screen the anti-leishmania anti-parasitic activity of novel potential therapeutic compounds, and have identified the flavonoid galganin as a potential prospect for further investigation. Because of its low cost and speed, this new assay represents a cost-effective approach for resource-constrained countries for the identification of new agents or to detect antimonial resistance prior to treatment.

Advances in our knowledge of the molecular biology of the Trypanosomatidae will continue to enhance our understanding of parasite ecology, evolution, and pathogenesis, and enable our search for potential chemotherapeutic agents and immunomodulatory approaches to treatment and management of these neglected diseases.

## Author Contributions

All authors listed have made a substantial, direct, and intellectual contribution to the work and approved it for publication.

## Funding

This study was partially funded by the UK Research and Innovation, Global Challenges Research Fund (EP/T003782/1), the University of Glasgow Small Grants Fund and Scottish Funding Council.

## Conflict of Interest

The authors declare that the research was conducted in the absence of any commercial or financial relationships that could be construed as a potential conflict of interest.

## Publisher’s Note

All claims expressed in this article are solely those of the authors and do not necessarily represent those of their affiliated organizations, or those of the publisher, the editors and the reviewers. Any product that may be evaluated in this article, or claim that may be made by its manufacturer, is not guaranteed or endorsed by the publisher.
